# Differences among families in craniofacial shape at early life-stages of Arctic charr (*Salvelinus alpinus*)

**DOI:** 10.1186/s12861-020-00226-0

**Published:** 2020-10-26

**Authors:** Samantha V. Beck, Katja Räsänen, Camille A. Leblanc, Skúli Skúlason, Zophonías O. Jónsson, Bjarni K. Kristjánsson

**Affiliations:** 1 Department of Aquaculture and Fish Biology, Hólar University, 551 Sauðárkrókur, Iceland; 2 Institute of Life- and Environmental Sciences, University of Iceland, Reykjavík, Iceland; 3 The Rivers and Lochs Institute, University of the Highlands and Islands, Inverness, UK; 4 Department of Aquatic Ecology, Eawag, Swiss Federal Institute of Aquatic Science and Technology, Dübendorf, Switzerland; 5 Institute of Integrative Biology, ETH Zürich, Zürich, Switzerland

**Keywords:** Developmental plasticity, Maternal effects, Ontogeny, Phenotypic divergence, Trophic morphology

## Abstract

**Background:**

Organismal fitness can be determined at early life-stages, but phenotypic variation at early life-stages is rarely considered in studies on evolutionary diversification. The trophic apparatus has been shown to contribute to sympatric resource-mediated divergence in several taxa. However, processes underlying diversification in trophic traits are poorly understood. Using phenotypically variable Icelandic Arctic charr (*Salvelinus alpinus*), we reared offspring from multiple families under standardized laboratory conditions and tested to what extent family (i.e. direct genetic and maternal effects) contributes to offspring morphology at hatching (H) and first feeding (FF). To understand the underlying mechanisms behind early life-stage variation in morphology, we examined how craniofacial shape varied according to family, offspring size, egg size and candidate gene expression.

**Results:**

Craniofacial shape (i.e. the Meckel’s cartilage and hyoid arch) was more variable between families than within families both across and within developmental stages. Differences in craniofacial morphology between developmental stages correlated with offspring size, whilst within developmental stages only shape at FF correlated with offspring size, as well as female mean egg size. Larger offspring and offspring from females with larger eggs consistently had a wider hyoid arch and contracted Meckel’s cartilage in comparison to smaller offspring.

**Conclusions:**

This study provides evidence for family-level variation in early life-stage trophic morphology, indicating the potential for parental effects to facilitate resource polymorphism.

**Supplementary information:**

**Supplementary information** accompanies this paper at 10.1186/s12861-020-00226-0.

## Background

Phenotypic variation is ubiquitous in natural populations, yet many of the processes and mechanisms underlying phenotypic variation are still little understood. The fitness consequences of phenotypic variation can be strong at early-life stages [1–5], yet early life-stage phenotypic variation is rarely considered in studies of evolutionary diversification. Fishes are the most species-rich clade of vertebrates, and offer an array of skull and jaw morphologies that reflect their ecological specialization [6–8]. One of the most famed examples of evolutionary diversification are the African rift-lake cichlids where adaptive radiation in feeding morphology has contributed to their rapid diversification [9–12]. If such variation in trophic structures is expressed already early in life, then individuals may be able to specialize on alternative resources, increasing phenotypic variation within a population [13], and thus providing fuel for natural selection. It is hypothesized that individual specialization to different diets may reduce intraspecific competition [14, 15], which can facilitate divergence of trophic morphology and the evolution of resource polymorphism (i.e. discrete intraspecific morphs that have diverged upon different resources) [16–19]. Ultimately, resource polymorphism may facilitate sympatric speciation [19–21] and provide valuable insight into evolutionary processes underlying diversification.

Glacial retreats following the last ice age have provided a window of opportunity to examine such processes in polymorphic Northern freshwater fishes, whereby their degree of phenotypic divergence often varies between different systems [22]. However, what initially generates phenotypic variation within populations is still poorly understood. By focusing on individual variation at very early life-stages (i.e. prior to the onset of feeding), this study aims to understand which factors promote variability in trophic morphology within a single population of Arctic charr (*Salvelinus alpinus*), and potentially shed light on the mechanisms underlying early stages of evolutionary diversification.

The extent of intraspecific divergence can be biased along both genetic and developmental lines of least resistance [23, 24], expanding upon existing phenotypic variation, but limited by available resources [25]. Importantly, the phenotype is often more malleable early in the development than later in life [23], because gene expression is more dynamic [26–29], can influence developmental trajectories (discussed in: [29–31]) and traits are not yet fixed. Studying early life-stage gene expression jointly with morphology can thus provide insight into developmental processes initiating phenotypic variation (e.g. [32]). Gene expression patterns have been linked to phenotypic divergence between different morphs in several taxa [31–36], but early life-stage variation in gene expression and morphology within morphs have been less well-studied. Particularly at early life-stages, variation in gene expression and morphology can be influenced by parental effects, for instance as differential distribution of maternal resources (i.e. egg size) which, in turn, can contribute to trophic specialization [33, 37, 38].

Parental effects – when the phenotype or performance of an individual is affected by the phenotype or environment of its parents [39] – are a common source of phenotypic variation at early life-stages [40, 41] and play a crucial role during early development [39]. The extent to which offspring phenotype is influenced by maternal effects can depend on multiple maternally transmitted factors such as yolk, mRNA transcripts or other cytoplasmic factors packaged in the egg by the mother during oogenesis [16, 41]. Studying early developmental stages can not only reveal the impact that parental effects might have on phenotypic variation of their offspring, but also gives insight into how phenotypes vary before exposure to external sources of environmental variation, such as diet. Importantly, variation in trophic morphology in developing embryos may reflect genetic and non-genetic parental effects and affect fitness of individuals when they start independent feeding.

The high propensity of Arctic charr for intraspecific diversity makes this species well suited for studying mechanisms that promote and/or precede the evolutionary origins of phenotypic diversity, especially associated with trophic polymorphism [42, 43]. Although several studies have examined associations between parental effects, gene expression and phenotypic variation between established morphs (see references above), which are sometimes visible at early-life stages [43, 44], there are relatively few (if any) studies examining such associations within a morph, or a population. Here we study a single morph of Arctic charr (Vatnshlíðarvatn brown [45];) to examine the association between early life-stage phenotypic variation (size and morphology), family (incl. Direct genetic and/or maternal effects) and gene expression at hatching (H) and first feeding (FF). Previously in this morph, we showed that early life-stage gene expression is very dynamic at early life-stages and that there is a correlation between offspring size and relative expression of two genes related to skeletal development and growth [29]: *Sgk1* showed a linear relationship with individual size, having higher expression in larger embryos at H, whereas *Star* was non-linear in relation to individual size. Using highly diverged benthic and pelagic morphs of Arctic charr, a previous study found that both of these genes – *Sgk1* and *Star* showed differential expression between the two morphs [33].

Here we combine our previous findings on dynamic early life-stage gene expression [29] with patterns of craniofacial morphology in the Vatnshlíðarvatn brown morph. We use acid-free double staining of cartilage and bone, coupled with geometric morphometrics, to test whether individuals from different families develop differently (i.e. resulting in different trophic morphologies at H and FF). We study variation among seven families, with family effects reflecting a combination of direct genetic and non-genetic parental effects. We studied eight growth-related genes (chosen from the literature based on their involvement during early development, [29]) and six genes related to skeletogenesis (based on findings in a previous study, [32]). Combining this previously collected gene expression data [29] with data on offspring morphology, we test the following predictions: 1) if there are genetic and/or parental effects in shape at early life-stages, we should see differences among families in craniofacial features; 2) if early life-stage phenotypic variation is related to genes involved in growth and skeletogenesis of trophic structures, offspring craniofacial shape should covary with the expression of the chosen candidate genes, and 3) if maternal investment (i.e. egg size) influences early life-stage phenotypes, female mean egg size and/or individual offspring size should correlate with offspring morphology.

## Results

### Craniofacial shape

**Fig. 1:**
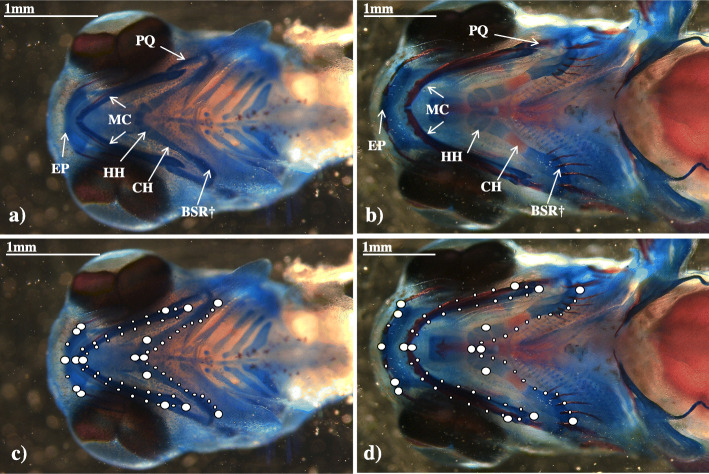
Craniofacial structures at two early life-stages (hatching and first feeding) in Arctic charr (*Salvelinus alpinus*). Ventral craniofacial bones and cartilages at hatching (**a**) and first feeding (**b**) of Arctic charr (*Salvelinus alpinus*) embryos from lake Vatnshlíðarvatn. EP, ethmoid plate; MC, Meckel’s cartilage; HH, hypohyal; CH, ceratohyal; BSR, branchiostegal rays; PQ, palatoquadrate. We refer to the combination of the HH, CH and BSR as the hyoid arch (HA). For timing and sequence of occurrence of these structures in Arctic charr see Kapralova et al. (2015). The 71 homologous landmarks used to quantify craniofacial shape change at hatching (**c**) and first feeding (**d**) can be divided into 17 fixed (larger dots) and 54 semi-landmarks (smaller dots). † BSR themselves were not considered for analyses but their attachment was incorporated as part of the HA

Measurement errors from digital images of morphology (Fig. 1) were calculated for replicates using the Procrustes ANOVA. Variation due to placement accounted for 6% at H and 10% at FF, whilst variation due to digitizing error was 3 and 6% at H and FF, respectively (Table S1). Once variation due to measurement error, as well as directional and fluctuating asymmetry, was accounted for, variation among individuals accounted for 73 and 70% of variation at H and FF, respectively (Table S1).

**Fig. 2:**
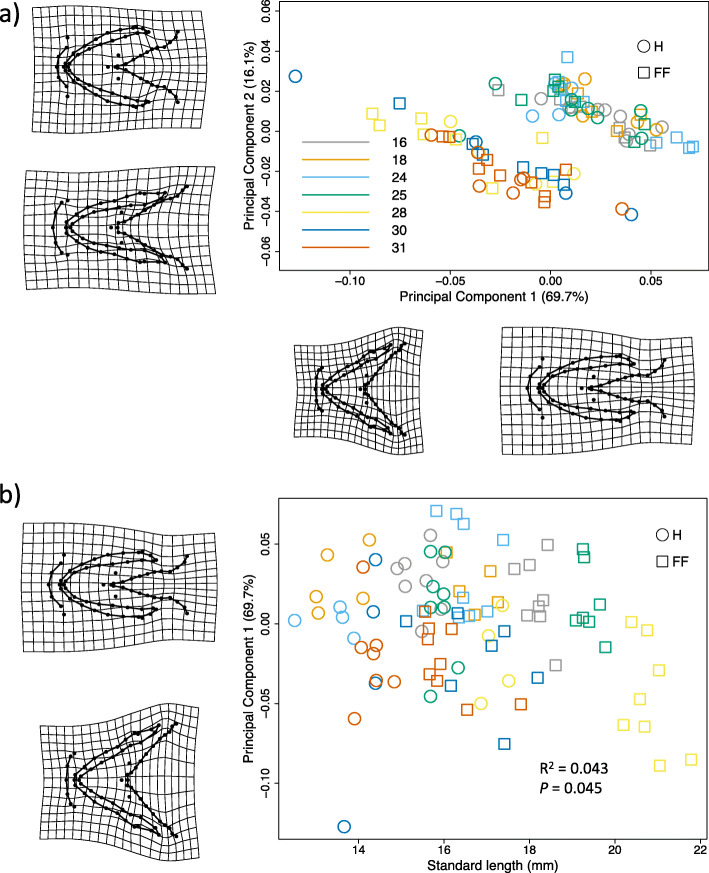
Family variation in craniofacial shape in Arctic charr (*Salvelinus alpinus*) at hatching and first-feeding. **a** Principal components analysis (PCA) plot showing craniofacial shape variation across families, and (**b**) the correlation between PC1 and offspring size (standard length, mm), at both hatching and first feeding in Arctic charr (*Salvelinus alpinus*) from lake Vatnshlíðarvatn. A total of 71 landmarks were used (see Fig. 1) and resulting deformation grids, with a 2x magnification, are presented at the extremes of both axes to facilitate the interpretation of shape change. Family identity (N offspring 5–9) is represented by different symbols

**Fig. 3:**
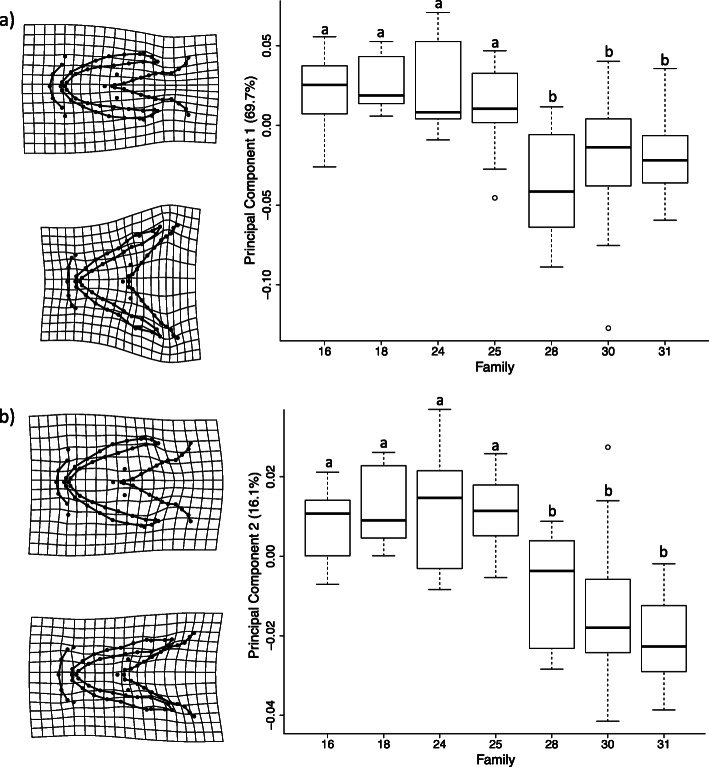
Mean family differences in offspring craniofacial shape in Arctic charr (*Salvelinus alpinus*). Principal co-ordinate 1 (PC1) from Fig. 2a explains the maximal variation in early life-stage craniofacial shape. **a** PC1 correlated against family identity (Z_6, 92_ = 4.00, *R*^*2*^ = 0.43, *P* = 0.001), with families 28, 30 and 31 differing from all other families based on least square means (*P* < 0.001), and **b**) PC2 and family (Z_6, 92_ = 4.70, *R*^*2*^ = 0.53, *P* = 0.001) with families 28, 30 and 31 also differing from all other families (*P* < 0.01). Deformation grids are presented at the extremes of both axes to facilitate the interpretation of shape change with a ×2 magnification

Morphological variation was analysed using geometric morphometrics, with craniofacial shape encompassing the hyoid arch and Meckel’s cartilage using 17 fixed 54 sliding semi-landmarks (Fig. 1). Only final models are shown (Table 1). Due to lack of any differences between the results of models with and without the effect of individual size (Table 1), we only report findings for those models with size included. Model 1 tested the effect of family and developmental stage on craniofacial shape. Family explained 40% of the variation in the craniofacial shape (Z_6, 92_ = 5.56, *R*^*2*^ = 0.40, *P* = 0.001), with no statistically significant effect of developmental stage (Z_1, 92_ = 0.51, *R*^*2*^ = 0.01, *P* = 0.313; Table 1). There were two clusters clearly visible in the Principal Components Analysis (PCA) plot (Fig. 2a; Fig. S1 with the effect of size removed), with PC1 explaining 69.7% and PC2 explaining 16.1% of variation in shape (Fig. 2a). For Model 1b, the correlation of PC1 and PC2 with offspring size and family within the same model resulted in a non-significant family effect, indicating that family effects were largely due to covariation with offspring size. As such, offspring size and family were tested separately alongside developmental stage. PC1 was correlated with both offspring size (Z_1, 92_ = 1.16, *R*^*2*^ = 0.04, *P* = 0.045) and family identity (Z_6, 92_ = 4.00, *R*^*2*^ = 0.43, *P* = 0.001), but not developmental stage (Table 1). However, when examining the relationship between offspring size and PC1 (Fig. 2b), offspring at H were clearly smaller and had a more-narrow hyoid arch than offspring at FF, which had a wider hyoid arch and contracted Meckel’s cartilage. When investigating family differences, those families that differed at H were not the same as those that differed at FF, and half-sib families (females 25, 28 and 31) did not cluster together away from full-sib families (Fig. 2a). All individuals from families 30 and 31 cluster exclusively in shape space, as well as most offspring from family 28 (with the exception of one individual). These three families showed a significantly wider hyoid arch and overall more contracted craniofacial shape along PC1 compared to the other families (*P* < 0.001, according to least square means; Fig. 3a), whilst for PC2 the same three families showed a more elongated phenotype in both the Meckel’s cartilage and hyoid arch (*P* < 0.01; Fig. 3b).

**Table 1:** Final Procrustes ANOVA models characterising early life-stage craniofacial shape in Arctic charr (*Salvelinus alpinus*) embryos at hatching (H) and first feeding (FF)

		Size								No Size						
			Df	SS	MS	Rsq	F	Z	P	Df	SS	MS	Rsq	F	Z	P
**Model 1**	H & FF	**Family**	**6**	**0.073**	**0.012**	**0.396**	**9.351**	**5.560**	**0.001**	**6**	**0.066**	**0.011**	**0.374**	**8.625**	**5.415**	**0.001**
		Dev. stage	1	0.001	0.001	0.007	0.997	0.506	0.313	1	0.001	0.001	0.006	0.791	0.244	0.416
		Residuals	85	0.110	0.001	0.600				85	0.109	0.001	0.614			
		Total	92	0.184						92	0.177					
**Model 1b**	PC1	**Off. size**	**1**	**0.005**	**0.005**	**0.043**	**4.057**	**1.158**	**0.045**							
		Dev. stage	1	0.000	0.000	0.004	0.360	0.161	0.540							
		Residuals	90	0.121	0.001	0.947										
		Total	92	0.128												
	PC1	**Family**	**6**	**0.056**	**0.009**	**0.434**	**10.875**	**3.997**	**0.001**	**6**	**0.050**	**0.008**	**0.406**	**9.951**	**3.921**	**0.001**
		Dev. stage	1	0.000	0.000	0.004	0.569	0.363	0.439	1	0.001	0.001	0.006	0.909	0.575	0.322
		Residuals	85	0.072	0.001	0.566				85	0.071	0.001	0.578			
		Total	92	0.128						92	0.123					
	PC2	Off. size	1	0.000	0.000	0.004	0.365	0.171	0.514							
		Dev. stage	1	0.000	0.000	0.000	0.013	−1.548	0.933							
		Residuals	90	0.029	0.000	0.994										
		Total	92	0.030												
	PC2	**Family**	**6**	**0.016**	**0.003**	**0.526**	**15.824**	**4.698**	**0.001**	**6**	**0.015**	**0.003**	**0.512**	**14.870**	**4.605**	**0.001**
		Dev. stage	1	0.000	0.000	0.000	0.025	−1.210	0.899	1	0.000	0.000	0.001	0.186	−0.232	0.653
		Residuals	85	0.014	0.000	0.471				85	0.014	0.000	0.488			
		Total	92	0.030						92	0.029					
**Model 2**	H & FF	Dev. stage	1	0.001	0.001	0.007	0.658	0.035	0.482							
		Egg size	1	0.006	0.006	0.031	2.905	1.579	0.061							
		Residuals	90	0.177	0.002	0.962										
		Total	92	0.184												
**Model 3**	H	Off. size	1	0.001	0.000	0.006	0.338	−0.763	0.779							
		**Family**	**6**	**0.029**	**0.005**	**0.367**	**3.213**	**2.914**	**0.003**	**6**	**0.028**	**0.005**	**0.368**	**3.198**	**2.903**	**0.002**
		Residuals	32	0.047	0.001	0.609				33	0.049	0.001	0.632			
		Total	39	0.078						39	0.077					
	FF	**Family**	**6**	**0.052**	**0.009**	**0.498**	**7.591**	**4.662**	**0.001**	**6**	**0.045**	**0.008**	**0.478**	**7.022**	**4.490**	**0.001**
		Residuals	46	0.053	0.001	0.502				46	0.049	0.001	0.522			
		Total	52	0.105						52	0.094					
		**Off. size**	**1**	**0.010**	**0.010**	**0.099**	**5.630**	**2.325**	**0.008**							
		Residuals	51	0.094	0.002	0.901										
		Total	52	0.105												
**Model 4**	H	Egg Size	1	0.000	0.000	0.002	0.083	−2.322	0.993							
		Residuals	38	0.078	0.002	0.998										
		Total	39	0.078												
	FF	**Egg size**	**1**	**0.010**	**0.010**	**0.096**	**5.391**	**2.212**	**0.014**							
		Residuals	51	0.095	0.002	0.904										
		Total	52	0.105												

Significant effects are indicated in bold. *Dev. stages* Developmental stages; *Off. Size* Offspring size; *Egg size* Mean egg size of each female; *Df* Degrees of freedom; *SS* Sum of squares; *MS* Mean square; *R*^*2*^ R-square; *F* F-value; *Z* Z-value; and *P P*-value of the significance of variables on influencing shape

**Fig. 4:**
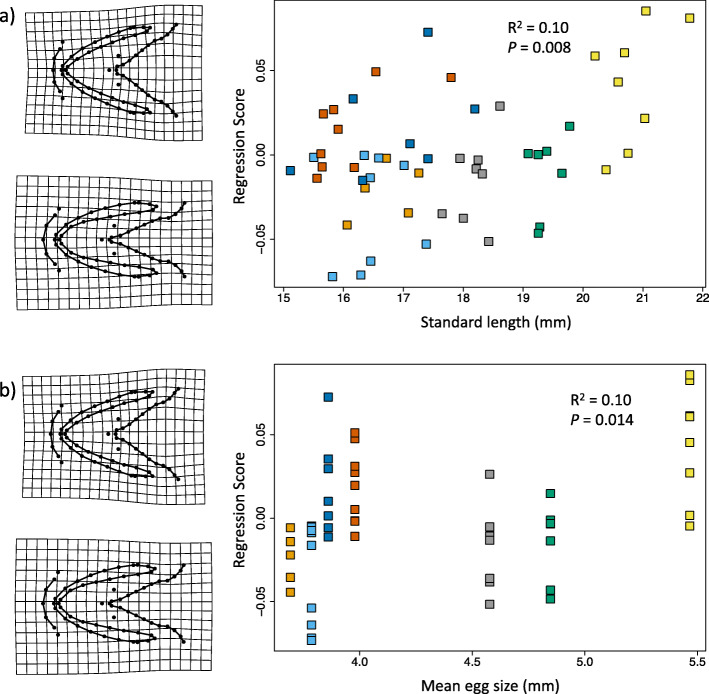
Relationship between craniofacial shape, offspring size and egg size in Arctic charr (*Salvelinus alpinus*) at first feeding. Procrustes regression of craniofacial shape of offspring at first feeding on: **a**) individual offspring size (standard length, mm); and **b**) mean female egg size (diameter, mm). Deformation grids at the extremes of both axes represent the amount of shape change according to offspring/egg size, with three-fold magnification

Model 2 investigated whether family mean egg size influenced craniofacial shape across developmental stages, both of which were found to have no effect (Table 1). For Model 3, we examined the effect of offspring size and family within each developmental stage. For H, offspring size and family were not confounded and could therefore be included within the same model, with family explaining 37% of variation in the shape of the hyoid arch and Meckel’s cartilage at H (Z_6, 39_ = 2.91, *R*^*2*^ = 0.37, *P* = 0.003), and no effect of offspring size (Table 1). Offspring size and family identity were confounded at FF and were therefore tested separately. Family explained 50% of the variation in craniofacial shape at FF (Z_6, 52_ = 4.66, *R*^*2*^ = 0.50, *P* = 0.001), whilst only 1% was due to offspring size 1% (Z_1, 52_ = 2.33, *R*^*2*^ = 0.10, *P* = 0.008), with larger offspring having a wider hyoid arch and a more contracted Meckel’s cartilage than smaller offspring (Fig. 4a).

Our final model, Model 4, examined whether there was a relationship between female mean egg size and craniofacial shape within each developmental stage. Only mean egg size at FF had a weak correlation with craniofacial shape (Z_1, 52_ = 2.21, *R*^*2*^ = 0.10, *P* = 0.014), the general pattern of which seems to show that offspring from females that produce larger eggs tend to have a wider hyoid arch and more contracted Meckel’s cartilage than those from females that produce smaller eggs (Fig. 4b).

### Covariance between gene expression and craniofacial shape

Variation between individuals/family in relative gene expression levels can be found in [29], where all genes showed dynamic expression patterns across post-fertilization, eyed stage, H and FF. To briefly summarize, *Mmp9* – a gene involved in osteogenesis – had the highest expression at H stage, whilst the only genes that had expression levels correlated with offspring size at H, were *Star* and *Sgk1*. Three genes involved in bone remodeling were found to increase at the onset of FF (*Ets2, Sparc* and *Timp2*), whilst there was a gradual increase throughout development for *Ctsk* and *Mmp9*, both of which play a role in ossification. The technical duplicates/per biological sample gave highly similar values as indicated by a very high correlation between the two technical replicates (*r* = 0.996, *P* < 0.0001, *N* = 1776; Fig. S2).

**Fig. 5:**
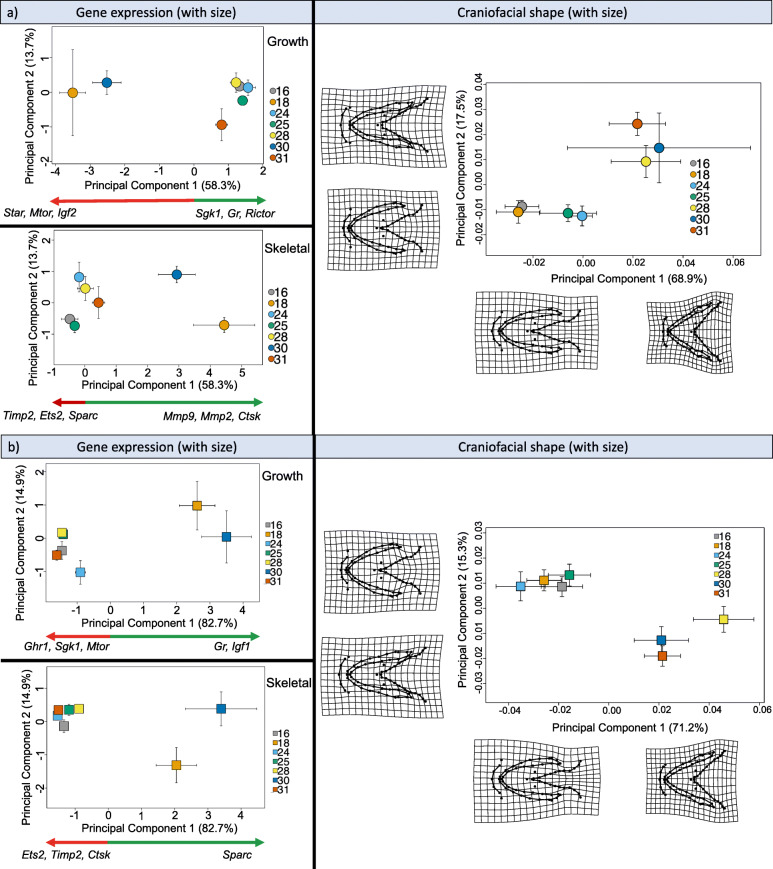
Craniofacial shape and gene expression in Arctic charr (*Salvelinus alpinus*) embryos at hatching and first feeding. Principal components analysis (PCA) plots showing both relative gene expression (for growth and skeletal related genes, modified [29]) and ventral craniofacial shape variation attributed to family in the brown Arctic charr morph from lake Vatnshlíðarvatn, at a) hatching (H), and b) first feeding (FF), with the effect of offspring size included (see Fig. S2 for PCA without the effect of size). Coloured shapes and associated standard error bars correspond to mean shape of offspring within a family. For gene expression only, green and red arrows show those genes indicated with the highest PCA loadings at the positive (green) and negative (red) extremes of the PC axes, with the top three genes (where applicable) listed from the highest to lowest loadings. Deformation grids at the extremes of both axes represent the amount of shape change, with two-fold magnification

Craniofacial shape and relative gene expression for both growth and skeletogenic genes did not covary at either H or FF stage (Fig. 5; Table S2; Fig. S3 for size-free shapes). Despite this lack of covariance, we demonstrate the loadings of genes that have driven divergence in gene expression between families towards the positive (green arrows) and negative (red arrows) extremes of the PC axis in Fig. 5 (see Fig. S4 for individual data). For gene expression only, families 18 and 30 cluster away from other families at both H and FF. Whereas, for craniofacial shape, families 28, 30 and 31 cluster together at H and FF.

## Discussion

This study found the effect of family in the “brown” Arctic charr morph from Lake Vatnshlíðarvatn to be a major source of early morphological variation in offspring craniofacial shape (i.e. the Meckel’s cartilage and hyoid arch) throughout early development. There was no evidence for covariance between craniofacial shape and relative expression of genes related to growth and skeletogenesis. Although developmental stage was not found to be significant, when examining the relationship between offspring size and PC1 of craniofacial shape, offspring at H were clearly smaller and have a more-narrow hyoid arch than offspring at FF (Fig. 2b). Within each developmental stage, only the craniofacial shape of offspring at FF correlated with offspring size and female mean egg size, with larger offspring and offspring from females with larger eggs having a wider hyoid arch and more contracted Meckel’s cartilage than smaller offspring and offspring from females with smaller eggs (Fig. 4). Understanding the way in which variation in structures associated with feeding during early development is generated, and whether such variation can promote craniofacial divergence, may shed light upon the possible mechanisms and processes involved in the early origins of evolutionary diversification.

### Family effects on craniofacial shape

Few studies have examined differences in morphology among families, despite their importance in creating variation and providing opportunities for the establishment of individual specialization, an important prelude to the evolution of resource polymorphism [13, 25]. Dietary specialization can often promote divergence of multiple ecologically distinct morphs, resulting from a process that occurs along a continuum of increasingly discrete variation: ranging from individuals that specialize upon different resources, to discrete resource morphs [13]. However, the source of this variation is less well-understood. This study found that craniofacial morphology of a single morph of Arctic charr was more variable between families than within families, with family differences seen in the hyoid arch and Meckel’s cartilage (Fig. 3). The involvement of the hyoid arch in the support and extension of the jaw suggests that offspring from different families may have the potential to differ in their efficiency for feeding on different types of prey [46, 47]. The differential ability to consume certain prey types can ultimately promote trophic divergence and resource polymorphism given the availability of alternate resources [48]. The hyoid arch is also involved in the respiratory cycle [49], whereby variation in its shape may indicate adaptive differences in respiratory needs. For example, benthic lake whitefish (*Coregonus clupeaformis*) from hypoxic benthic environments have larger gills, whilst pelagic planktivorous morphs tend to have larger gill lamellae surface areas [50, 51], each of which might correlate with a wider or a narrower hyoid arch, respectively. These family differences therefore represent an important source of variation to consider for the evolution of resource polymorphisms, especially given the results from this study which demonstrate the strong influence of family on offspring that have been reared in common-garden conditions. Understanding whether similar family differences are seen in offspring in the wild, and the extent to which their fine-scale phenotypic variation interacts with environmental conditions, will determine the importance of family differences in providing the initial variation for intraspecific diversification to occur.

### Developmental stage differences

In general, fishes undergo both morphological and ecological changes during early life-stages due to changes in functional or structural requirements [47, 52]. Using ventral views of Arctic charr heads only, we found no differences in craniofacial shape due to developmental stage alone (Table 1), but offspring at H were smaller and had a more-narrow hyoid arch than those larger FF offspring (Fig. 2b). Few studies examine craniofacial shape differences within a single population, and instead focus of differences between morphs and/or species [33, 53–55]. Such studies may miss any differences between individuals, a potential source of phenotypic variation that might promote divergence in the wild. Our study demonstrates the potential of offspring size for driving inter-individual differences in the studied craniofacial shape structures across development, particularly in different females. Future studies examining internal craniofacial shape variation between individuals should increase the number of landmarks, but most importantly characterise craniofacial development in 3D, capturing ventral, dorsal and lateral changes in morphology (e.g. reviewed in Hallgrimsson et al. [56]). Such an approach will increase the likelihood of detecting fine-scale variation in the shape of embryos and fish larvae and may further reveal how the head is shaped over both developmental and evolutionary time.

### Influence of gene expression on craniofacial shape

Phenotypic variation is often the result of changes in gene expression levels in response to environmental conditions, which has the potential to enhance evolutionary divergence when exposed to natural selection [57–59]. Although such differences may not always be apparent at the phenotypic level, analysis of gene expression provides an opportunity to uncover hidden phenotypes that may later become of ecological relevance [60]. Here we found no evidence to suggest that there is any covariance between relative expression of genes related to growth and skeletogenesis and morphology. Such lack of covariance might be a result of a time lag between the expression of certain genes and their translation into offspring phenotype. How and when certain genes are expressed and translated into phenotypic differences is uncertain, and may occur either much earlier or much later in development [26, 61]. This study was also designed to eliminate plastic responses to the environment during early development, therefore any fine-scale variation in feeding structures that would otherwise be enhanced during the feedback between morphology, food, habitat choice and their interaction with gene expression [62], would not be detected.

### Effects of individual offspring size and mean female egg size on offspring morphology

Our results show that craniofacial morphology was correlated with individual offspring size (standard length) and mean egg size (diameter) per female at FF only, with larger offspring and offspring from females with larger eggs having a wider hyoid arch than smaller offspring (Fig. 4; Table 1). The influence of a maternally-mediated trait (egg size) on trophic morphology at FF can potentially have an impact on what diet is available to her offspring [63]. Furthermore, offspring-size related morphological differences in trophic structures can influence what food resources are available to offspring with more narrow or wider trophic structures (from smaller or larger offspring, respectively: Fig. 4), thus influencing trophic specialisation - a major driver of morphological divergence [7, 64]. However, such findings are weak and also confounded with family effects, with offspring from different families also differing in size. Nevertheless, this study highlights the potential role that offspring size and egg size may play in promoting intraspecific diversification,

## Conclusion

Variability in trophic structures have the potential to restrict what food resources are available to offspring when they start feeding, yet our understanding of what generates this initial variation is incomplete. Here we demonstrate family differences in the shape of feeding and respiratory structures that have been linked to the evolutionary divergence of sympatric morphs [43, 51, 65, 66]. Variation in feeding structures during FF may facilitate divergence in resource use in those habitats where intraspecific competition is high, and alternate resources are available [14]. It is this fine-scale variation in feeding structures that has the potential to elicit initial divergence in diet, which may then be further emphasised through plasticity, and possibly the generation of discrete resource-morphs [19, 67–69].

Our understanding of the mechanisms and processes forming and maintaining biodiversity are limited, despite anthropogenic effects being one of the major underlying causes for the dramatic loss in todays’ biodiversity [70, 71]. Studies documenting phenotypic and genetic differences in morphs or populations that differ along a gradient of evolutionary diversification are needed to be able to advance our understanding of mechanisms that may promote or precede the evolutionary origins of biodiversity.

## Methods

### Study system

We used Arctic charr of the so called ‘brown’ morph from lake Vatnshlíðarvatn, Iceland (65°51´693 N, − 19°63′ 536 W). Vatnshlíðarvatn is a physically simple, small (70 ha) and shallow (average depth 2–3 m) lake [45]. Arctic charr is the only fish species in the lake and has diverged into two weakly divergent morphs (silver and brown [45, 72];). The brown morph shows extensive variation in egg size and egg diameter, and standard length of embryos at H and FF are strongly correlated (*N* = 12 families, H: *R*^*2*^ = 0.96, N offspring = 201, FF: *R*^*2*^ = 0.97, N offspring = 168; both *P* < 0.001, Leblanc et al. unpublished data).

The clutches and rearing protocols used in this study are described in [29]. In short, mature females (*N* = 7) and males (*N* = 5) were caught using gill nets in September 2014. Fertilised eggs were allowed to water-harden before transport. Each female was mated to a single male (i.e. full sib families), with a subset of (*N* = 3) females sharing the same male (i.e. half-sib families). This design causes variation in the relatedness of offspring and does not allow us to fully disentangle direct genetic from maternal effects but was used to minimize unsuccessful crosses. We henceforth use the term ‘family effects’. All adults were euthanized by lethal cranial concussion in the field after stripping and before transport back to Verið aquaculture facilities. Age of females was estimated by reading otoliths, as described by Tsinganis [73].

Fish were grown under the ethics permit of Verið aquaculture station and in accordance with Icelandic law. Experiments in this study were conducted within the ethical framework of the 3Rs (Replacement, Reduction and Refinement) as follows: 1) alternatives to the experimental procedures carried out by this research study were not available and thus the use of fish in this study was considered essential; 2) only the minimal number of fish required for the experiments were used, and all experiments were conducted on early-life stages and ended prior to independent feeding; and 3) animal welfare levels were high as experiments were conducted in an experienced aquaculture station with excellent expertise in fish husbandry practices.

Embryos were reared in the laboratory in darkness in mesh cages in a shelf incubator system with a constant flow of 95% recycled water, at a temperature of 3.5–4.5 °C. Before eye stage, a subset of at least 15 eggs were measured per family to determine mean family egg size. Developmental timing was tracked with an accumulative temperature estimate (degree days, DD [74];). Embryos were randomly collected at: 1) H, when individuals have emerged from the egg but still rely on nutrition from the yolk sac (at 461 ± 3.11 DD); and 2) FF, when individuals begin feeding (at 658 ± 12.58 DD, see Table 2 for sample summary). Samples for the gene expression (*N* = 82) and craniofacial shape (*N* = 94) analyses were collected at the same time for each family (Table 2). All embryos were euthanized using 600 ppm of 2-phenoxyethanol [75], a widely approved method [76]. All individuals (eggs, or left-side of H and FF embryos) were digitally photographed (Canon EOS 650D) with a mm scale, and eggs measured four times each to obtain average diameter as a measure of egg size, and once for measurement of standard length, SL (for H and FF stages [77];), to the nearest 0.01 mm, using the program Fiji [78].

**Table 2:** Sampling numbers and measurements of 7 Arctic charr (*Salvelinus alpinus*) females (♀) and their offspring (Off)

Family (ID)	♀ FL (cm)	♀ age	♀ egg size (mm)	Male ID	Mortalities	Hatching (H)			First Feeding (FF)		
						Off. N Stain	Off. N GE	DD	Off. N Stain	Off. N GE	DD
16	17.6	5	4.6 ± 0.28	34	7	8	7	464	8	5	657
18	14.5	5	3.8 ± 0.29	36	43	5	6	456	5	7	654
24	15.2	6	3.8 ± 0.24	33	5	4	5	462	9	4	632
25	18.6	6	4.8 ± 0.37	35	12	8	6	460	7	5	659
28	20.6	8	5.4 ± 0.46	35	8	4	6	463	8	8	665
30	14.7	4	4.3 ± 0.54	32	6	5	6	464	7	7	666
31	14.2	4	4.0 ± 0.29	35	19	7	4	458	9	6	670

Arctic charr Embryos from lake Vatnshlíðarvatn were sampled across two developmental stages (hatching (H) and first feeding (FF)) for gene expression (GE) and staining of bone and cartilage. Measurements ± standard deviation (SD) *FL* Fork length; *DD* Degree days; *N* Sample size; *GE* Gene expression

### Gene expression data collection

Gene expression data was taken from Beck et al. [29], which uses offspring from the same dataset to characterise inter-individual variation in the expression of genes related to growth and skeletogenesis. A candidate gene approach using genes found to exhibit expression differences due to either family, developmental stage or offspring size in a previous study, was selected over an analysis of the whole transcriptome in order to maximise the number of individuals per family (Beck et al.*,* in prep. [79];). Briefly, a total of 14 candidate genes were chosen: six target genes involved in promoting trophic skeletogenesis (*Timp2, Ets2, Sparc, Ctsk, Mmp2* and *Mmp9*) [80] and display differential expression in divergent Arctic charr morphs from lake Þingvallavatn [33]; whilst the remaining eight candidate genes (*Star, Igf1, Igf2, Gr, Mtor, Sgk1, Rictor* and *Ghr1*) were chosen using a literature search based on evidence of their involvement in promoting growth, especially during early embryonic development [81–89].

Total RNA was extracted from the whole embryo at H using TRI reagent (Sigma–Aldrich, St Louis, MO) after homogenizing tissues in the Bead Beater (Biospec). The same RNA extraction methods were applied to offspring at FF. However, due to their larger size, individuals were decapitated behind the pectoral fin and only the RNA extracted from the head was included in this study as most of the phenotypic variation in Arctic charr is related to trophic morphology. As in Beck et al. [29], RNA was precipitated using isopropanol, washed with ethanol and air-dried. The RNA pellet was resuspended in RNase-free water and treated with DNase I (New England, Biolabs, Ipswich, MA) to remove any contaminating DNA. A subset of extracted RNAs were electrophoresed on agarose gels to test RNA quality. Single stranded cDNA was synthesized from 1 μg of total RNA, using the High Capacity cDNA Reverse Transcription kit (Applied Biosystems, Foster City, CA) according to the manufacturer’s protocol. cDNA was consequently diluted in nuclease-free water in preparation for qPCR.

Primers [29] were designed using an assembled Arctic charr transcriptome [90] and exon boundaries mapped to *Salmo salar* orthologs from the salmonid species database [91]. Primers spanned at least one exon boundary and were selected based upon their short amplicon size (< 250 bp). RT-qPCR was performed on 96-well PCR plates on a 7500 Real-Time PCR System (Applied Biosystems) using 2x Fermentas Maxima SYBR Green qPCR Master Mix (Fisher Scientific, Pittsburgh, PA, USA), with a final reaction volume of 10 μl. For family-level variation we had 4–8 individuals (replicates) per family, and a total of 7 families that represent biological replicates. Each biological sample was run in technical duplicates with a non-template control in each run for each gene. Primer efficiencies were calculated [29] and both RT-qPCR and differential mRNA gene expression calculations for each target gene performed according to [33], using two validated early developmental Arctic charr reference genes, *Actb* and *Ef1a* [92]. Reference genes were normalised by randomly selecting one individual within each developmental stage as a calibrator sample. Relative expression quantities (RQ) were calculated according to [33].

### Staining of craniofacial elements and digitization

For analyses of morphology, individuals were fixed in 4% paraformaldehyde (PFA) and stained with Alizarin red for bone, and Alcian blue for cartilage, using a modified acid-free double staining protocol [44, 93], see Additional file 1. Based on a previous study [53], as well as preliminary trials, we selected craniofacial elements that were clearly visible at both H and FF stages (Fig. 1; for an overview on all craniofacial structures in developing embryos and time of appearance [53]).

All samples were stained simultaneously within each developmental stage and the same staining solutions were used across both developmental stages to ensure as much consistency as possible. Once stained, individuals were placed in a petri-dish containing 2 and 3% transparent methylcellulose (Appendix S1) for H and FF, respectively, to allow easy maneuverability during photography and to ensure that the embryos lay flat. Individuals were photographed ventrally using a HD digital microscope camera (LEICA MC170 HD) mounted on a stereomicroscope (LEICA M165 C), with a scale set for each photograph. Each individual was photographed twice, each time removing and re-positioning the fish to account for measurement error due to placement. The two sets of photos per individual were duplicated so each individual was represented by a total of four photos: two photos to account for placement error, and two photos to account for digitizing error. Photos of disfigured individuals (*N* = 8) were removed and remaining photos randomized before digitization. To capture the ventral craniofacial shape of developing Arctic charr and to enable comparisons between H and FF stages, we chose 17 fixed landmarks and 54 sliding semi-landmarks (to increase effect sizes [94]) that were homologous between developmental stages, and placed on ventral surfaces of individuals in tpsDig2 v.2.31 ( [95]; Fig. 1). Landmarks were placed on trophic structures, such as the hyoid arch, Meckel’s cartilage and ethmoid plate. Semi-landmarks were placed at set equal distances along a curve between two fixed landmarks, using tpsDig2. The relative measurement error due to the differences in placement of individuals for photos, as well as digitising error, was evaluated for each developmental stage using Procrustes ANOVA [96] in MorphoJ [97], before taking the average of all photos per individual to remove such measurement errors.

#### Statistical analyses

### Gene expression

Analyses were performed on log_2_ transformed RQ values and differences in gene expression across stages and families can be found in [29].

### Geometric morphometrics for craniofacial shape

All morphometric analyses were conducted in R v.3.3.2 [98] using the R package *geomorph* v.3.0.3 [99]. Procrustes shape residuals were obtained using a generalized Procrustes analysis [100], which optimally superimposes landmarks according to location, size and orientation. The resulting Procrustes residuals were then used for analysis of object symmetry [96], using the function *bilat.symmetry* to average landmarks across the line of symmetry to remove variation due to side, as well as averaging our replicates.

All analyses were conducted both with and without the effect of offspring size. For size-free shapes, residuals from the relationship between offspring size and morphology were obtained using *procD.lm.* The influence of developmental stage (H and FF) and family effects, as well as their interaction, on offspring craniofacial shape was determined using ANOVA from *procD.lm* (Model 1)*.* As *procD.lm* uses Type1 sum-of-squares, the position of the last variable was alternated in sequential regressions to obtain an unbiased estimate of the proportion of shape variance attributable to each variable [101]. A PCA was conducted on Procrustes shape residuals to visualize shape changes across families and developmental stage by plotting the first two axes from the PCA, whilst also showing morphological variation at the extremes of those axes in relation to the average morphology using the function *plotTangentSpace*. The first two PC axes explaining the most variation in craniofacial shape were then correlated with individual offspring size (standard length, mm), family and developmental stage (Model 1b), and significant relationships plotted. Data on egg size was only available as family means (Table 2). Therefore, family and mean egg size were not included within the same model. For Model 2 we tested whether female mean egg size had an effect on offspring craniofacial shape across developmental stages.

Further analyses were then conducted within each developmental stage: Model 3 examined the effect of offspring size on morphology, with family included as a random effect when the inclusion of both family and offspring size were not confounded. In cases where offspring size and family were confounded, they were tested separately; and finally, Model 4 determined the extent that mean female egg size had on craniofacial shape of offspring.

### Gene expression and craniofacial shape

To enable comparison of gene expression and craniofacial shape, we used family means within each data set (gene expression and shape at H and FF stages; *N* = 7) to determine the extent of their covariance. Using family means was necessary as data could not be collected on the same individuals for both gene expression and shape. Two-block partial least squares (PLS [102];) analysis was used to assess whether craniofacial shape (Procrustes residuals) covaried with the relative expression of genes related to skeletogenesis and/or growth within each developmental stage (due to the significant difference in gene expression between H and FF [29]).

A singular value decomposition is obtained from the two covariance matrices (craniofacial shape and relative gene expression), whereby the resulting PLS axes are uncorrelated, and where the first axis explains maximal covariation between both blocks (gene expression and shape [101, 102];). The amount of covariation between the two blocks was measured using a multivariate correlation coefficient (r_PLS_: [103]), with associated *P*-values based on 10,000 permutations under the null hypothesis of independence between both blocks of variables.

Individual PC plots for both gene expression and craniofacial shape at each developmental stage were used to facilitate our understanding of how differences in gene expression levels (for both sets of genes) may correspond with differences in craniofacial shape. These PC plots will be displayed with gene loadings from the PLS analyses to determine the strength of genes in any covariance between gene expression and craniofacial shape.

## Supplementary information

**Additional file 1.** Appendix S1. Acid-free double staining protocol adapted from Walker & Kimmel [93] and Kapralova [44].

**Additional file 2 Table S1.** Procrustes ANOVA of shape differences between replicated craniofacial measurements of Arctic charr embryos. Calculation of measurement error due to digitising error, placement of individuals as well as calculating variation due to fluctuating and directional asymmetry.

**Additional file 3 Table S2.** Partial least square (PLS) results from analysis of covariance between craniofacial shape (block 1) at two early life-stages (H, hatching and FF, first feeding) and relative expression of 14 candidate genes related to craniofacial development (block 2) in Arctic charr (*Salvelinus alpinus*).

**Additional file 4 Figure. S1**. Principal components analysis (PCA) plot showing size-free craniofacial shape variation across families of Arctic charr (*Salvelinus alpinus*) from lake Vatnshlíðarvatn at hatching and first feeding. A total of 71 landmarks were used (see Fig. 1) and resulting deformation grids, with a 2x magnification, are presented at the extremes of both axes to facilitate the interpretation of shape change. Family identity (N offspring 5–9) is represented by different symbols.

**Additional file 5 Figure S2**. A graph showing the correlation between technical duplicates of critical threshold (Ct) values (representing 14 genes related to growth and skeletal development) per biological sample of Arctic charr (*Salevlinus alpinus*) at hatching and first feeding (*r* = 0.996, *P* < 0.0001, *N* = 1776).

**Additional file 6 Figure S3.** Principal components analysis (PCA) plots showing both relative gene expression (for growth and skeletal related genes) and ventral size-free craniofacial shapes attributed to family in the brown Arctic charr morph from lake Vatnshlíðarvatn, at a) hatching (H), and b) first feeding (FF). Coloured shapes and associated standard error bars correspond to mean shape of offspring within a family. For gene expression only, green and red arrows show the top three genes (listed from highest to lowest) with the highest PCA loadings driving divergence towards the positive (green) and negative (red) extremes of the PC axes. Deformation grids at the extremes of both axes represent the amount of shape change, with two-fold magnification.

**Additional file 7 Figure S4.** Principal components analysis (PCA) plots showing both relative gene expression (for growth and skeletal-related genes) and ventral craniofacial shape variation attributed to family in the brown Arctic charr morph from lake Vatnshlíðarvatn at hatching (H) and first feeding (FF). a) H with offspring size; b) H without offspring size; c) FF with offspring size; and d) FF without offspring size. Coloured shapes and associated standard error bars correspond to mean shape of offspring within a family. For gene expression only, green and red arrows show the top three genes (listed from highest to lowest) with the highest PCA loadings driving divergence towards the positive (green) and negative (red) extremes of the PC axes. Deformation grids at the extremes of both axes represent the amount of shape change, with two-fold magnification.

## Data Availability

Gene expression data included in this study is available in the Dryad repository, 10.5061/dryad.653q97g. The morphological dataset used and analysed during the current study are available from the corresponding author on reasonable request.

## Abbreviations

3D: Three-dimensional
ANOVA: Analysis of variance
cDNA: Complementary DNA
DD: Degree days
DNA: Deoxyribonucleic acid
FF: First feeding
H: Hatching
HD: High-definition
mRNA: Messenger RNA
mm: Millimetres
N: Sample size
PC: Principal component
PCA: Principal component analysis
PFA: Paraformaldehyde
PLS: Two-block partial least squares
ppm: Parts per million
qPCR: Quantitative polymerase chain reaction
RNA: Ribonucleic acid
RQ: Relative expression quantities
RT-qPCR: Real-time quantitative polymerase chain reaction
SL: Standard length
μg: Microgram
