# Supplementary material for: Differences among families in craniofacial shape at early life-stages of Arctic charr (Salvelinus alpinus)
Source: BMC Dev Biol. 2020 Oct 26;20:21. doi: 10.1186/s12861-020-00226-0 (PMC7586659; doi:10.1186/s12861-020-00226-0)
Supplement: Supplementary file 1 — Additional file 1. Appendix S1. Acid-free double staining protocol adapted from Walker & Kimmel [93] and Kapralova [44]. [file 12861_2020_226_MOESM1_ESM.docx]

#### Fixation

Embryos or larvae were fixed in 4% paraformaldehyde and stored in 100% Methanol at -20.

1. Transfer the embryos to 100% (or 96%) Ethanol and rock for 10 mins

#### Staining

Embryo (for larvae, skip to step 4):

1. Add 1 ml of acid-free Alcian blue, and 10ul of Alizarin red to each microfuge tube, and rock overnight

#### Bleaching

1. Remove the staining solution and add 1ml of water, mixby inversion and remove
2. Add 1 ml of the bleaching solution and incubate for **20 minutes** at room temperature with the lids open. ## no rocking
3. Add 1ml of water and mix by inversion

#### Clearing

1. Add 1 ml of a solution of 25% glycerol and rock at room temperature for 3 days
2. Replace with 1 ml of 50% glycerol and rock for 3
3. Replace with 1 ml of 80% glycerol and rock for 3

#### Photography

1. Prepare a solution of methylcellulose (2% for H, 3% for FF)

#### Storage

In a solution of 80% glycerol at 4°C.

#### Preparation of solutions

#### *Alcian blue stock (0.4% Alcian blue in 70% ethanol)*

For 100ml:

1. 0.04g of Alcian blue powder
2. Dissolve in 50% ethanol, incubated and stirred at 37°C
3. Add 95% ethanol and water to obtain final concentration

#### *Alcian blue for sampling (part A)*

1. Take 25ml of dH20 and dissolve 60 mM MgCl_2_
2. Add 5ml of Alcian blue stock solution (0.4%)
3. Add 70ml 95% ethanol

#### *Alizarin red (part B)*

0,5% Alizarin red S powder dissovled in dH20.

Acid- free double stain solution contains 10µl of 0.5%Alizarin red (*part B*) and 1 ml of Alcian blue (*part A*) which are mixed just prior to staining.

#### Bleaching solution

Mix equal amounts of 3% H_2_O_2_ and 2% KOH to give final concentration 1,5% H_2_O_2_ and 1% KOH

#### Methylcellulose (2% for 100ml)

1. Heat ~30mL 1xPBS
2. Place ~70mL 1xPBS in a beaker on ice
3. Add 2g of methylcellulose powder to 1xPBS and stir
4. Add ice cold 1xPBS (70mL) to solution. Stir on ice.
5. Make aliquots and store at 4 °C
