# Supplementary material for: Differences among families in craniofacial shape at early life-stages of Arctic charr (Salvelinus alpinus)
Source: BMC Dev Biol. 2020 Oct 26;20:21. doi: 10.1186/s12861-020-00226-0 (PMC7586659; doi:10.1186/s12861-020-00226-0)
Supplement: Supplementary file 2 — Additional file 2 Table S1. Procrustes ANOVA of shape differences between replicated craniofacial measurements of Arctic charr embryos. Calculation of measurement error due to digitising error, placement of individuals as well as calculating variation due to fluctuating and directional asymmetry. [file 12861_2020_226_MOESM2_ESM.docx]

| Hatching (H) | | | | | |
| --- | --- | --- | --- | --- | --- |
| Effect | **SS** | **MS** | **Df** | **F** | ***P*** |
| Individual | 0.5948 | 0.0002 | 2960 | 4.89 | **<.0001** |
| Side | 0.0240 | 0.0003 | 74 | 7.9 | **<.0001** |
| Ind*Side | 0.1217 | 0.0000 | 2960 | 5.08 | **<.0001** |
| Placement | 0.0479 | 0.0000 | 5920 | 4.05 | **<.0001** |
| Replicate | 0.0239 | 0.0000 | 11988 |  |  |
| Total | 0.8123 |  | 23902 |  |  |
| First feeding (FF) | | | | | |
| Individual | 1.0598 | 0.0002 | 6512 | 7.11 | **<.0001** |
| Side | 0.0615 | 0.0008 | 74 | 36.31 | **<.0001** |
| Ind*side | 0.1490 | 0.0000 | 6512 | 2.07 | **<.0001** |
| Placement | 0.1441 | 0.0000 | 13024 | 3.18 | **<.0001** |
| Replicate | 0.0912 | 0.0000 | 26196 |  |  |
| Total | 1.5056 |  | 52318 |  |  |

Individual, variation attributable to actual differences between individuals after the effects of object symmetry and measurement error had been removed; Side, i.e. directional asymmetry, the average difference between the two sides of symmetry; Ind*Side, i.e. fluctuating asymmetry, the variability between left and right sides among individuals; Placement, variation due to placement of individuals in petri-dish; Replicate, variation due to digitising error.

SS, sum of squares. MS, mean squares. Df, degrees of freedom. F, F-statistic.
