# Supplementary material for: Differences among families in craniofacial shape at early life-stages of Arctic charr (Salvelinus alpinus)
Source: BMC Dev Biol. 2020 Oct 26;20:21. doi: 10.1186/s12861-020-00226-0 (PMC7586659; doi:10.1186/s12861-020-00226-0)
Supplement: Supplementary file 3 — Additional file 3 Table S2. Partial least square (PLS) results from analysis of covariance between craniofacial shape (block 1) at two early life-stages (H, hatching and FF, first feeding) and relative expression of 14 candidate genes related to craniofacial development (block 2) in Arctic charr (Salvelinus alpinus). [file 12861_2020_226_MOESM3_ESM.docx]

|  | **Craniofacial shape (Block 2)** | | | | | | | |
| --- | --- | --- | --- | --- | --- | --- | --- | --- |
|  | Size | | | | No size | | | |
|  | H | | FF | | H | | FF | |
| **Relative gene expression (Block 1)** | r_PLS_ | *P* | r_PLS_ | *P* | r_PLS_ | *P* | r_PLS_ | *P* |
| Growth | 0.71 | NS | 0.22 | NS | 0.39 | NS | 0.15 | NS |
| Skeletal | 0.47 | NS | 0.15 | NS | 0.51 | NS | 0.16 | NS |

NS, non-significant; r_PLS,_ multivariate correlation coefficient; *P*, *P*-values based on 10,000 permutations under the null hypothesis of independence between both blocks of variables.
