# Supplementary material for: Differences among families in craniofacial shape at early life-stages of Arctic charr (Salvelinus alpinus)
Source: BMC Dev Biol. 2020 Oct 26;20:21. doi: 10.1186/s12861-020-00226-0 (PMC7586659; doi:10.1186/s12861-020-00226-0)
Supplement: Supplementary file 4 [file 12861_2020_226_MOESM4_ESM.docx]

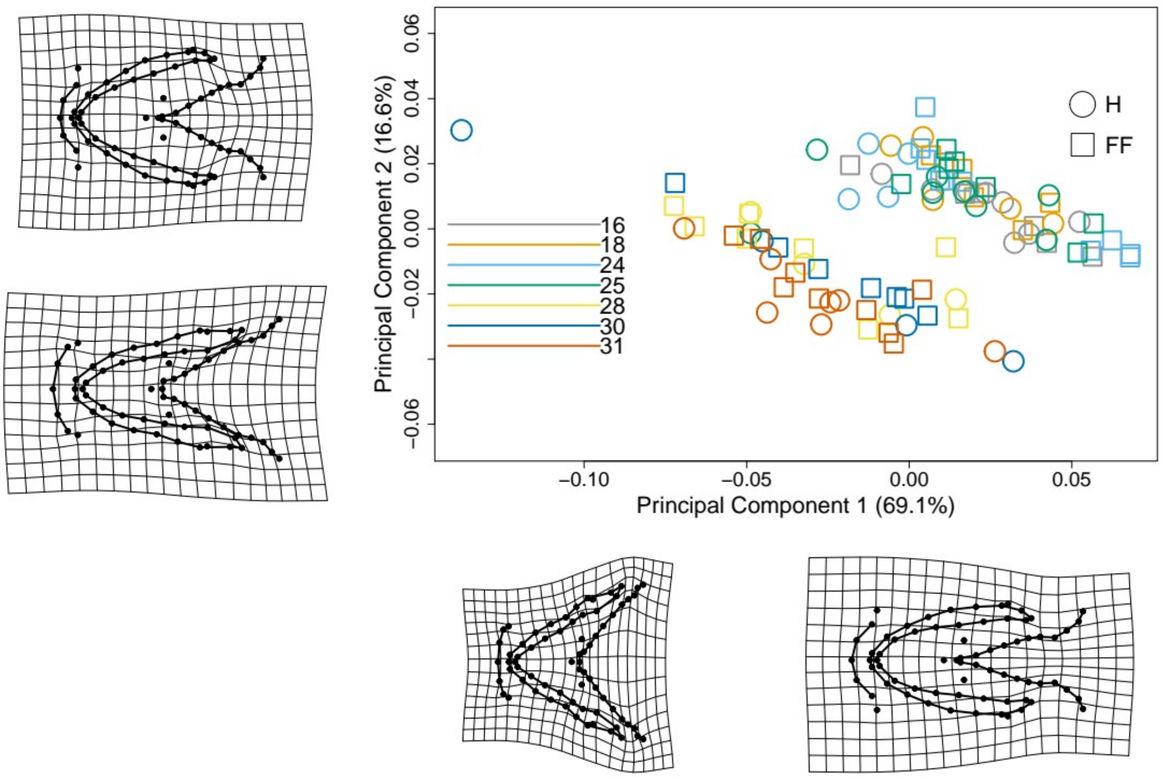

0.06
0.04
0.02
0.00
−0.02
−0.06
Principal Component 2 (16.6%)
−0.10
−0.05
0.00
0.05
Principal Component 1 (69.1%)
H
FF
16
18
24
25
28
30
31
