# Supplementary material for: Differences among families in craniofacial shape at early life-stages of Arctic charr (Salvelinus alpinus)
Source: BMC Dev Biol. 2020 Oct 26;20:21. doi: 10.1186/s12861-020-00226-0 (PMC7586659; doi:10.1186/s12861-020-00226-0)
Supplement: Supplementary file 5 [file 12861_2020_226_MOESM5_ESM.docx]

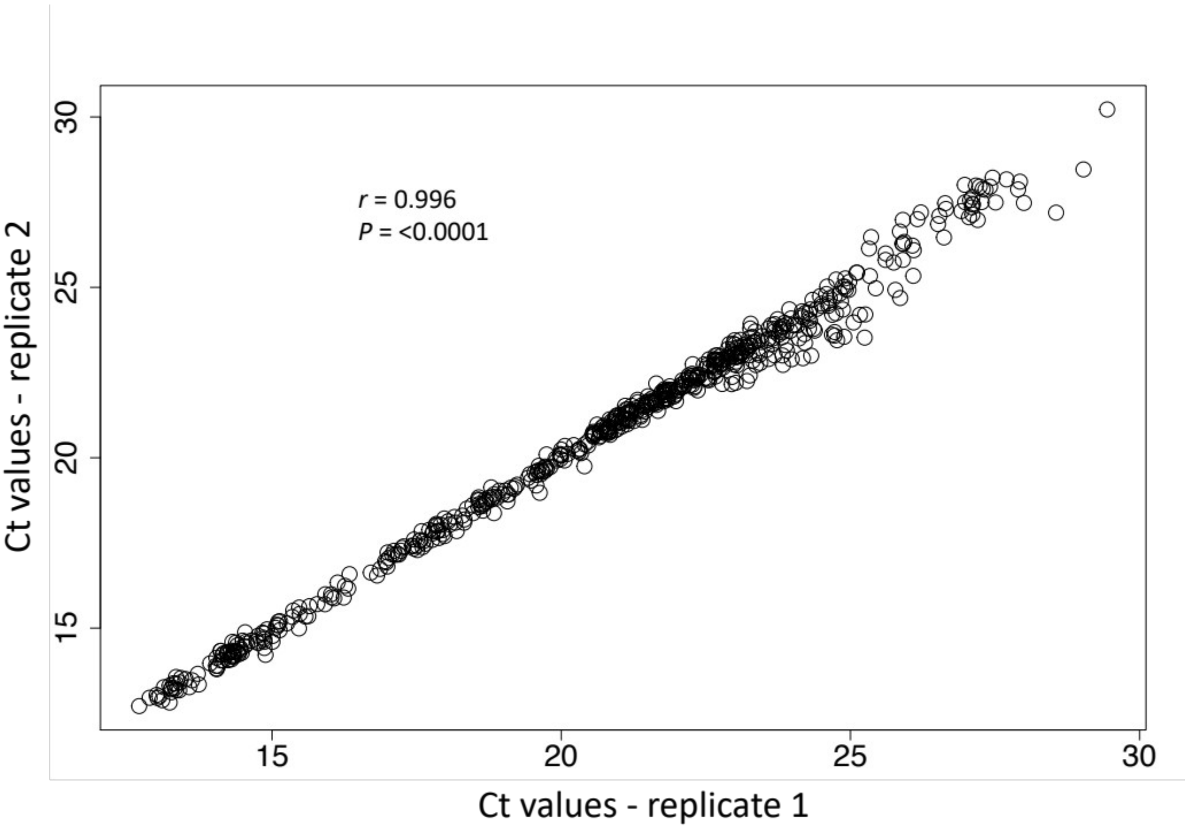

r = 0.996
P = <0.0001
Ct values - replicate 2
Ct values - replicate 1
15
20
25
30
15
20
25
30
