# Supplementary material for: Differences among families in craniofacial shape at early life-stages of Arctic charr (Salvelinus alpinus)
Source: BMC Dev Biol. 2020 Oct 26;20:21. doi: 10.1186/s12861-020-00226-0 (PMC7586659; doi:10.1186/s12861-020-00226-0)
Supplement: Supplementary file 6 [file 12861_2020_226_MOESM6_ESM.docx]

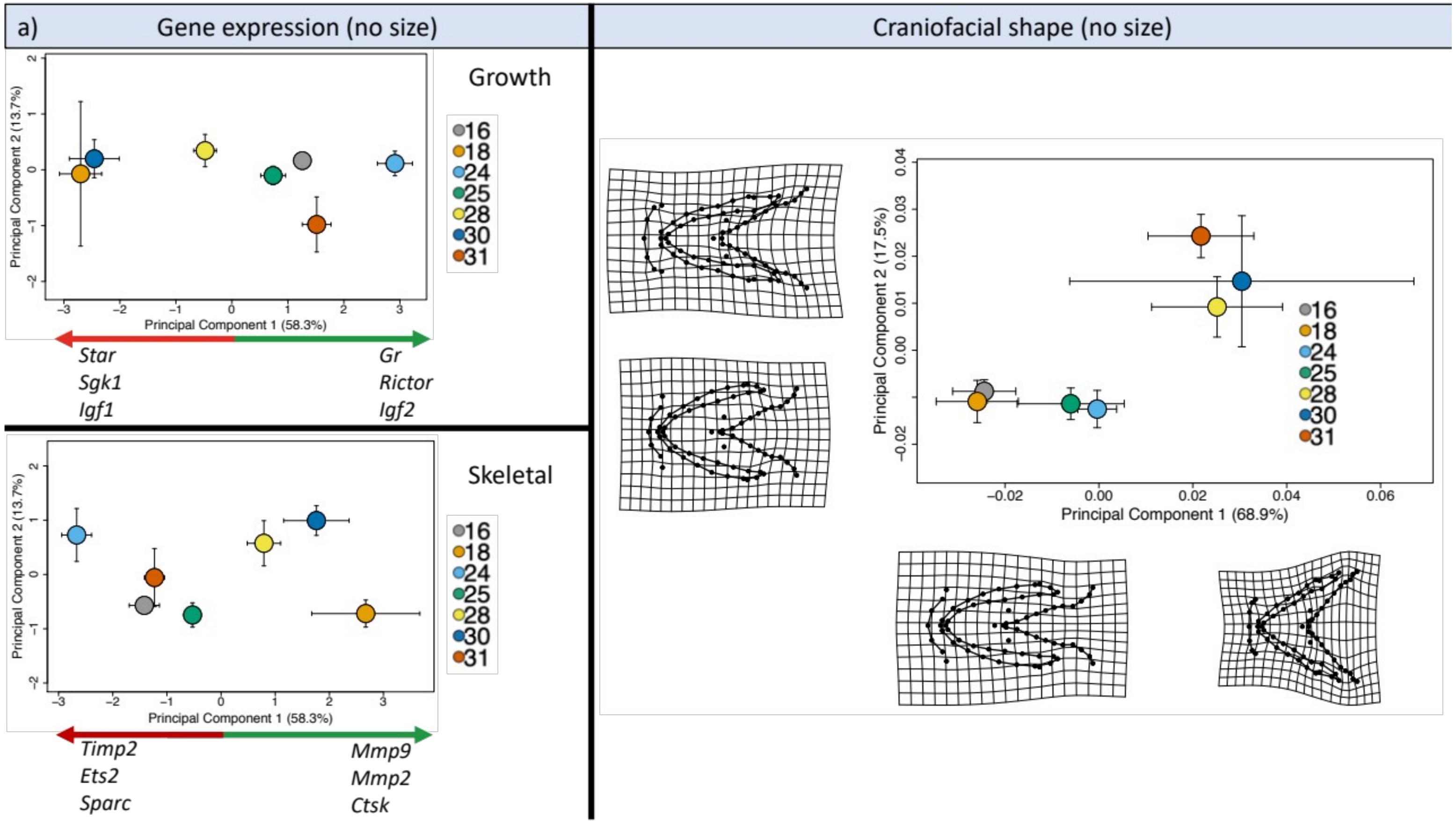
a)
Gene expression (no size)
Craniofacial shape (no size)
Growth
Principal Component 2 (13.7%)
Principal Component 1 (58.3%)
16
18
24
25
28
30
31
Star
Sgk1
Igf1
Gr
Rictor
Igf2
Skeletal
Principal Component 2 (13.7%)
Principal Component 1 (58.3%)
Timp2
Ets2
Sparc
Mmp9
Mmp2
Ctsk
Principal Component 2 (17.5%)
Principal Component 1 (68.9%)
