# Supplementary material for: Differences among families in craniofacial shape at early life-stages of Arctic charr (Salvelinus alpinus)
Source: BMC Dev Biol. 2020 Oct 26;20:21. doi: 10.1186/s12861-020-00226-0 (PMC7586659; doi:10.1186/s12861-020-00226-0)
Supplement: Supplementary file 7 [file 12861_2020_226_MOESM7_ESM.docx]

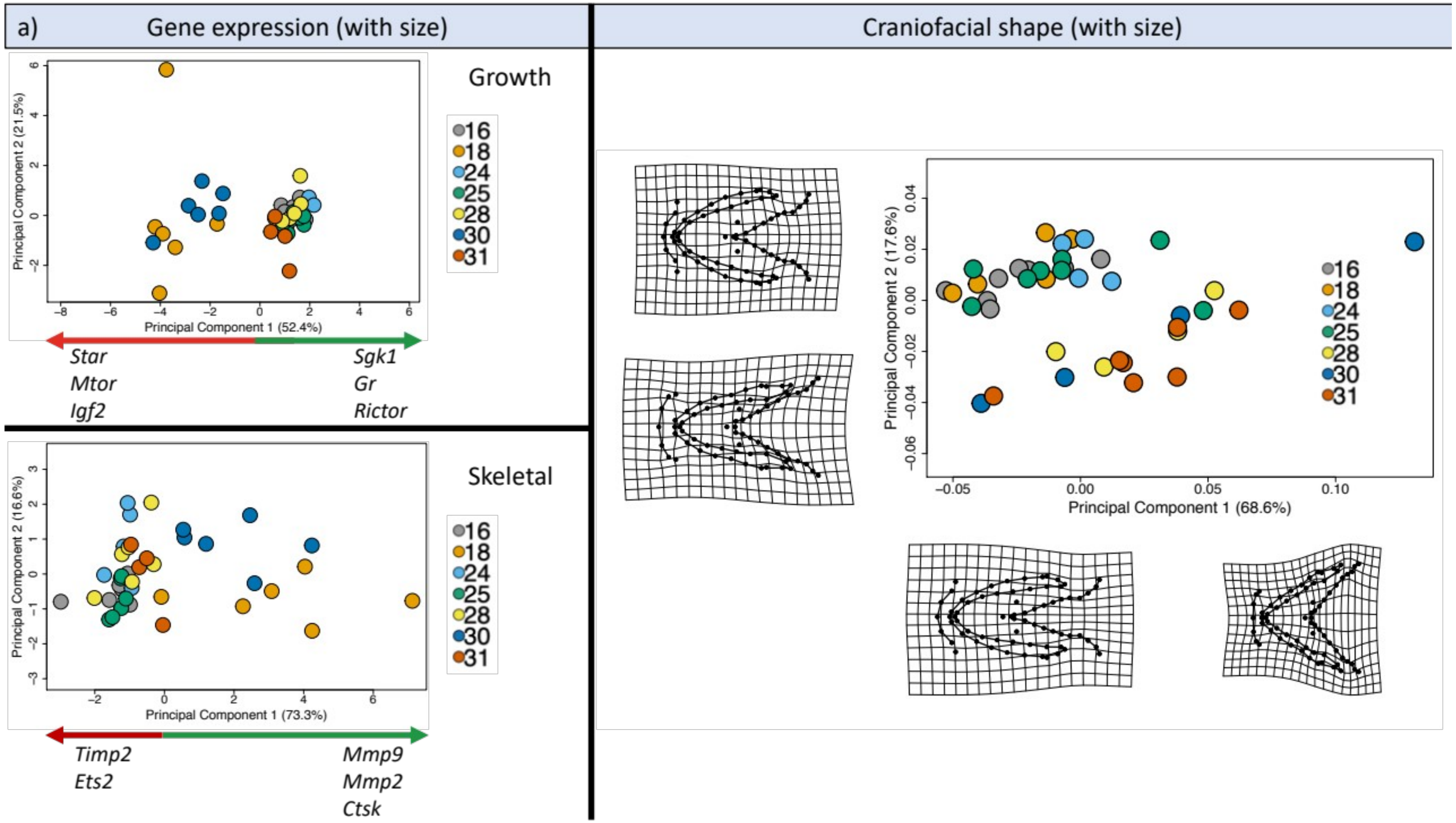
a)
Gene expression (with size)
Craniofacial shape (with size)
Growth
Principal Component 1 (52.4%)
Principal Component 2 (21.5%)
16
18
24
25
28
30
31
Star
Mtor
Igf2
Sgk1
Gr
Rictor
Skeletal
Principal Component 1 (73.3%)
Principal Component 2 (16.6%)
Timp2
Ets2
Mmp9
Mmp2
Ctsk
Principal Component 1 (68.6%)
Principal Component 2 (17.6%)

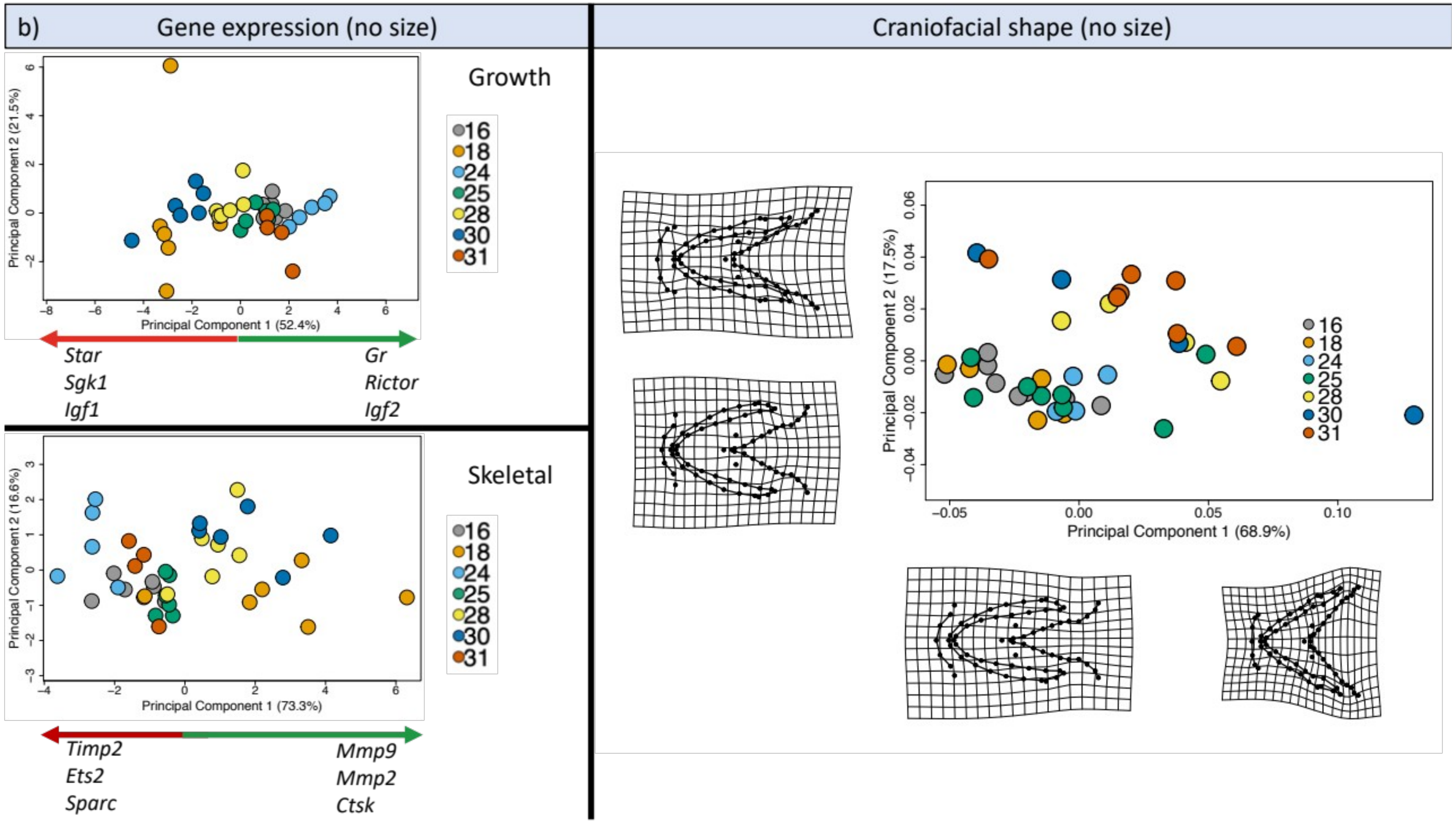

b)
Gene expression (no size)
Craniofacial shape (no size)
Growth
Principal Component 1 (52.4%)
Principal Component 2 (21.5%)
16
18
24
25
28
30
31
Star
Sgk1
Igf1
Gr
Rictor
Igf2
Skeletal
Principal Component 1 (73.3%)
Principal Component 2 (16.6%)
Timp2
Ets2
Sparc
Mmp9
Mmp2
Ctsk
Principal Component 1 (68.9%)
Principal Component 2 (17.5%)

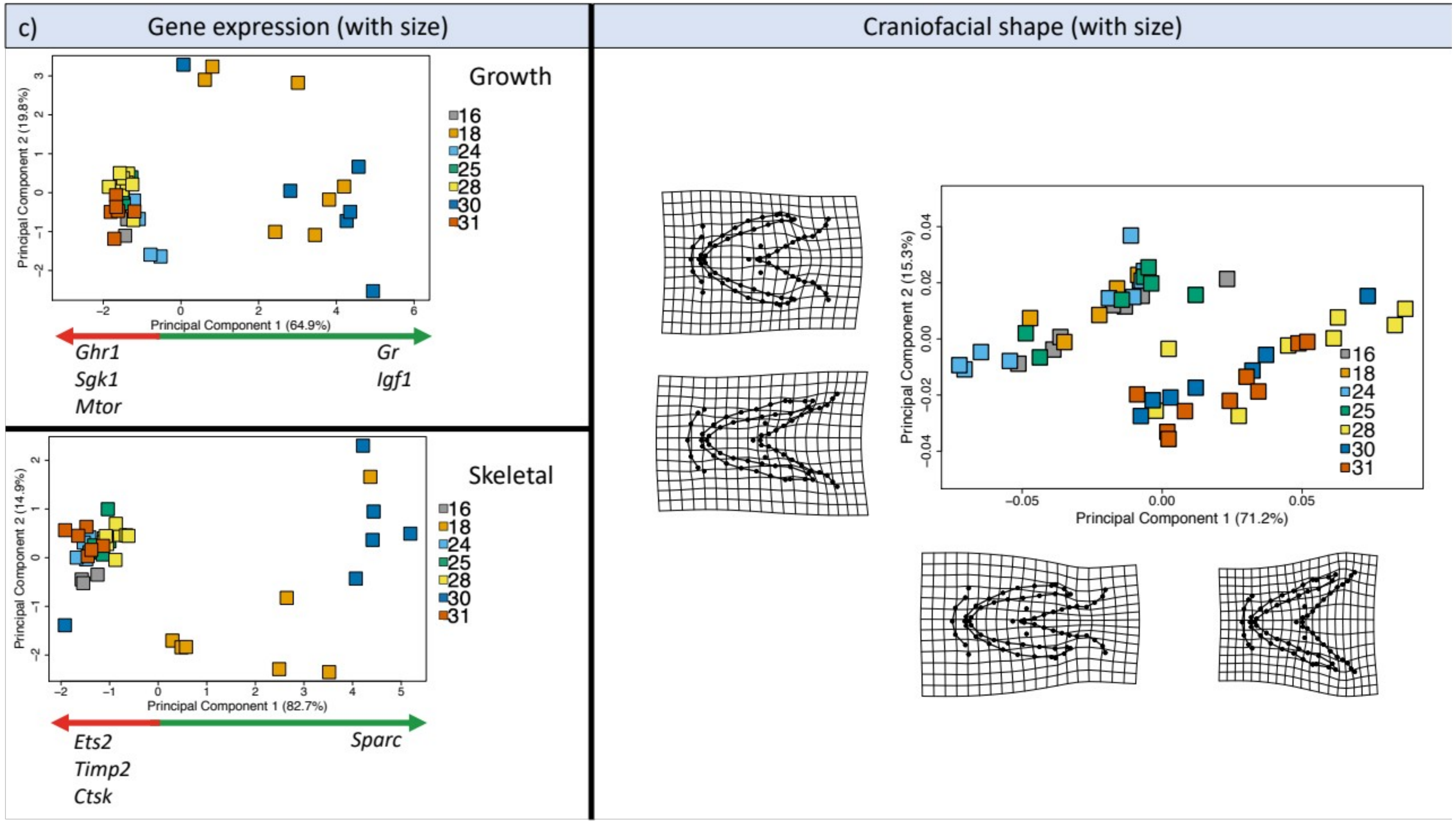
c)
Gene expression (with size)
Craniofacial shape (with size)
Growth
Principal Component 2 (19.8%)
Principal Component 1 (64.9%)
16
18
24
25
28
30
31
Ghr1
Sgk1
Mtor
Gr
Igf1
Skeletal
Principal Component 2 (14.9%)
Principal Component 1 (82.7%)
Ets2
Timp2
Ctsk
Sparc
Principal Component 2 (15.3%)
Principal Component 1 (71.2%)

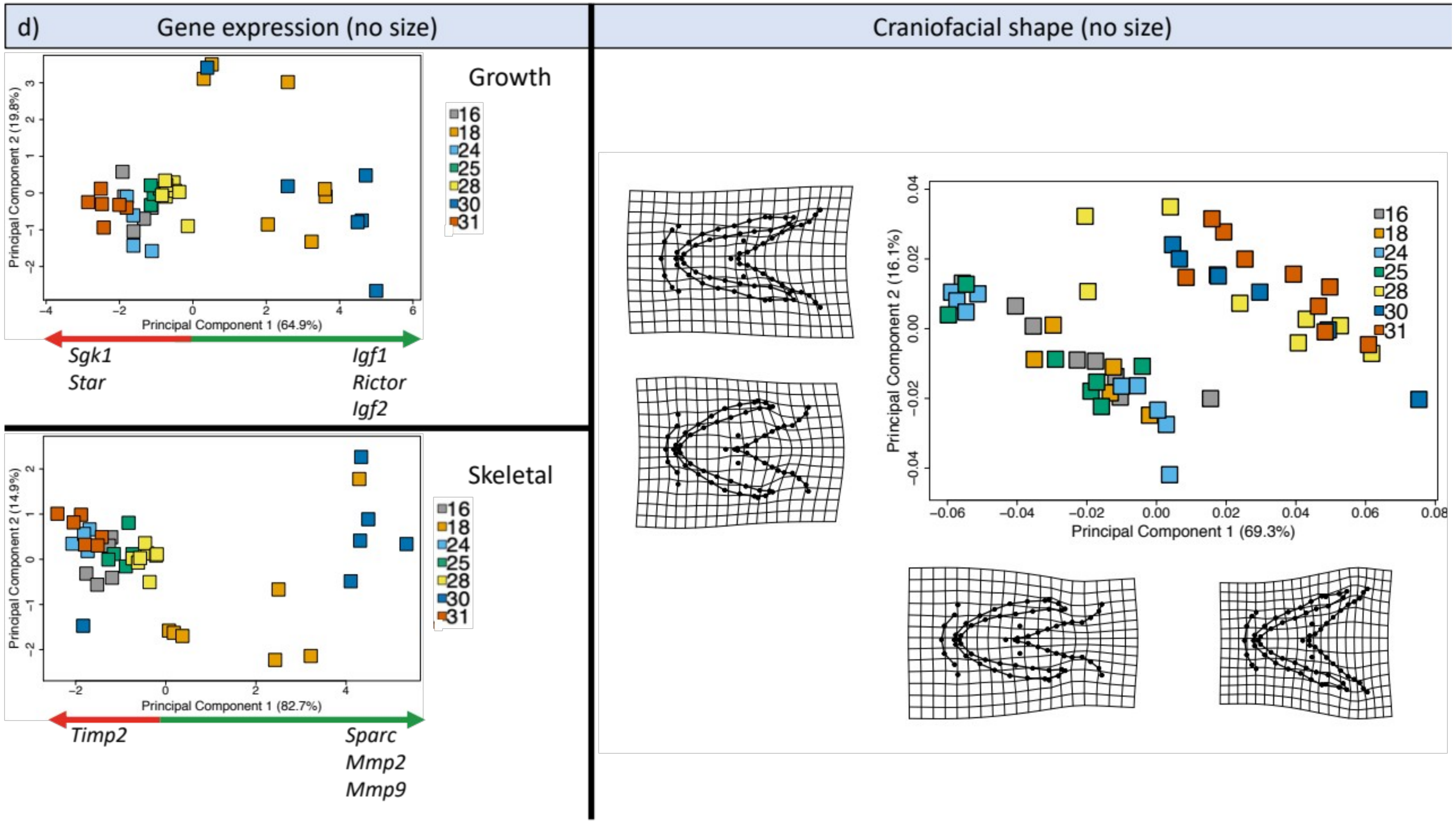

d)
Gene expression (no size)
Craniofacial shape (no size)
Growth
Principal Component 1 (64.9%)
Principal Component 2 (19.8%)
Sgk1
Star
Igf1
Rictor
Igf2
Skeletal
Principal Component 1 (82.7%)
Principal Component 2 (14.9%)
Timp2
Sparc
Mmp2
Mmp9
Principal Component 1 (69.3%)
Principal Component 2 (16.1%)
16
18
24
25
28
30
31
